# Chemical Composition and Elements Concentration of Fillet, Spine and Bones of Common Carp (*Cyprinus carpio*) in Relation to Nutrient Requirements for Minerals

**DOI:** 10.3390/ani14091311

**Published:** 2024-04-27

**Authors:** Agnieszka Kaliniak-Dziura, Piotr Skałecki, Mariusz Florek, Monika Kędzierska-Matysek, Paweł Sobczak

**Affiliations:** 1 Department of Quality Assessment and Processing of Animal Products, Faculty of Animal Sciences and Bioeconomy, University of Life Sciences in Lublin, Akademicka 13, 20-950 Lublin, Poland; piotr.skalecki@up.lublin.pl (P.S.); mariusz.florek@up.lublin.pl (M.F.); monika.matysek@up.lublin.pl (M.K.-M.); 2 Department of Food Engineering and Machines, Faculty of Production Engineering, University of Life Sciences in Lublin, Głęboka 28, 20-612 Lublin, Poland; pawel.sobczak@up.lublin.pl

**Keywords:** proximate composition, mineral content, *Cyprinus carpio*, bone tissue, fish by-products

## Abstract

**Simple Summary:**

Examination of the nutritional mineral composition of carp (*Cyprinus carpio*) tissues revealed that both edible (fillet) and inedible parts of fish (spine and bones) can be good sources of compounds with functional or beneficial influence on human health. Due to the high mineral content, carp bones and spine can be used as a natural calcium source in food and feed or as a supplement. Therefore, *C. carpio* bone by-products could be utilized taking into account the principles of a circular economy and can be even exploited by the food industry on a larger scale, facilitating better promoting by-products of this widely cultured fish species.

**Abstract:**

The aim of the study was to compare the content of major components, selected elements and heavy metals in the fillet, spine and bones of a carp (*Cyprinus carpio*). Moreover, the extent to which a prepared portion of carp tissue (100 g of fillet and 10 g of carp spine or bones) met the requirements for analyzed elements in adults (women and men) and children was calculated. The proximate composition (total protein, total lipid, ash, moisture) and mineral content of the fish samples were determined. The nutrient composition presented fluctuations among the different tissues. Moisture was the main constituent in the fillet and in the spine with 77.8% and 56.0%, respectively, whereas in bones, the main ingredient was ash (36.2%). All carp tissues were good sources of protein, with 16.5%, 21.0% and 17.0% in spine, bones and fillet, respectively. The most abundant main elements were the potassium in the fillet (4005 mg kg^−1^) and calcium in the bones (116,463 mg kg^−1^). The most abundant trace elements were iron in fillet and zinc in bones and spine. Carp meat can be considered a safe foodstuff in terms of concentrations of Hg, Pb and Cd, as the levels of these contaminants were less than FAO and European Commission maximum guidelines.

## 1. Introduction

Aquatic foods are increasingly recognized for their key role in food security and nutrition, not just as a source of protein, but also as a unique and extremely diverse provider of essential omega-3 fatty acids and bioavailable micronutrients. Production of aquatic animals in 2020 was more than 60% higher than the average in the 1990s, considerably outpacing world population growth, largely due to increasing aquaculture production. The consumption of aquatic foods is also higher than ever—about 20.2 kg per capita in 2020—more than double the total consumption rate 50 years ago [1].

The carp (*Cyprinus carpio*) is one of the most widely cultured species in the world. Its annual production exceeds 4 million tonnes, which makes it the fourth most important aquaculture species, with high economic value [1]. In Central and Eastern Europe, pond aquaculture carp is by far the most cultivated fish [2]. Its high importance in aquaculture is probably due to a fast growth rate, easy cultivation and high feed efficiency ratio [3]. In Poland, which is one of Europe’s key carp producers [4], traditional carp ponds are an important part of human cultural heritage with high relevance for biodiversity [5].

Carp is mainly available for the consumer as a whole fish, considered as too big and difficult to prepare [6] and cannot compete with highly processed and much more convenient fish products made of different fish species. To increase the range of available carp products, many processors search for alternatives, e.g., the production of skinless fillets [7]. Such processing, however, generates considerable amounts of fish remains, e.g., head, gills, intestines, trimmings, bones and skin, that remain underused and/or unexploited. These remains of the fish are commonly called by-products and may form up to 15% of fish weight following gutting and scaling or even up to 70% after filleting [8,9].

By-products were traditionally considered to be of low value or as a problem and were used as feed for farmed animals, as fertilizers or discarded [8]. These products, however, may be a potential source of many healthy and biologically active compounds, which have significant potential to supply new and valuable food ingredients [10]. For example, bones, which constitute approximately 10–15% of total fish biomass [11], can serve as good sources of essential minerals [12,13,14].

Fish, obviously, cannot be eaten with bone in its natural state, as it cannot be chewed or digested [15]. Therefore, fish bones or carcasses are usually used for the preparation of fish soups and gelatins [16]. Bones from whole fish and remains can also be used as a raw material for health products and as ingredients in, as previously mentioned, feeds for aquaculture [11], with a positive effect on feed efficiency and growth compared to traditional diets [17]. Utilizing fish bones as a food additive would be an alternative way to fortify food products with calcium, as well as to promote nutritional and functional properties [18].

Food fortification is identified as one of the most effective strategies for complement micronutrient deficiencies [19]. Calcium fortified products are likely to play an important role in helping the consumers achieve an adequate Ca intake, especially for persons with a low intake of dairy products [20]. The most widely used products for fortification are rice, wheat, or maize flour, salt, sugar, milk, oils and fats [21]. Food products are commonly fortified using commercial salts such as tricalcium phosphate, calcium carbonate, calcium lactate and calcium citrate. However, natural calcium contained in fish bones can be comparably accepted by the consumers and even more bioavailable than most calcium salts [22,23].

Information about the chemical composition of fish bones is important in understanding the physiological role of bone tissue, but may be also useful in assessing the possibility of use of bone-rich fish by-products from the processing industry. Therefore, the knowledge of nutrients composition of the fish tissues, especially of any inedible parts, may contribute to increasing the value of by-products as well as to solving the problem of fish waste management [11,24]. Unfortunately, few data are given on the chemical composition of fish bones, especially of *Cyprinidae* fishes. There are, in fact, some works presenting the chemical composition of cyprinid bone powder [25,26]; however, they may not be quite comparable with studies focused on fish bone composition, as fish bone powder may contain lower quantities of organic materials such as fat and protein and have higher purity and ash content than fish frames [17]. Therefore, the aim of the present research was to compare the content of major components and the concentration of selected elements and heavy metals in fillet, spine and bones of common carp (*C. carpio*). The results were used to calculate the extent to which a prepared serving of carp tissue (100 g of fillet and 10 g of carp spine or bones) met the requirements for analyzed minerals in children and adults (women and men).

## 2. Materials and Methods

### 2.1. Material

The study material comprised 15 commercial carps (*C. carpio*) aged 3+, which were kept a certified fish farm with a low-intensity system. Fish were maintained in earth ponds placed in Lublin Province (Poland) in winter 2022. The feed of the fish was mainly natural food (benthos, zooplankton) with additional grain feeding (wheat, barley and rye). After catching, the fish were delivered to the farm facility as quickly as possible, then electrically stunned by trained fish farm workers and immediately killed by cutting the spinal cord. The average body weight of the carps was 1479 ± 231 g.

Fish, after killing, were immediately transported in a passive travel fridge, maintained at the temperature of 2 °C (±2 °C) and delivered to the laboratory within 2 h following capture. Fish were submitted for pre-treatment including descaling, gutting and heading. From the remaining carcass, the edible and inedible parts were separated, i.e., the fillet (muscle tissue without skin), which was used as a control group and the vertebral column (spine) and fish bones (ribs).

To prepare the spines and bones for analysis, they were microwaved at a power of 800–1200 W for 3–6 min according to a patented method [27] that facilitates the removal of muscle tissue remaining on the skeleton.

### 2.2. Determination of Proximate Composition

Moisture content was determined by drying (103 °C) in a Memmert UF30 universal oven (Schwabach, Germany) according to PN-ISO 1442:2000 [28]; total ash by incineration (at 550 °C) using a Heraeus M110 laboratory muffle furnace (Hanau, Germany) according to PN-ISO 936:2000 [29]; total protein content (N × 6.25) by the Kjeldahl method using the Büchi Speed Digester K-436 (Flawil, Switzerland) and the Büchi Distillation Unit B-324 (Flawil, Switzerland) according to PN-A-04018/Az3:2002 [30]; and fat content by the Soxhlet method (with n-hexane as solvent) using the Büchi Extraction System B-811 (Flawil, Switzerland) in accordance with PN-ISO 1444:2000 [31]. The samples were analyzed in duplicate. The energy value of 100 g of fillet (kJ/100 g) was calculated from the total protein and fat content, using physical energy equivalents (23.64 kJ for 1 g protein, 39.54 kJ for 1 g fat) [32]. The nutritional quality index (NQI) was calculated according to Hansen et al. [33] for protein and fat, adopting reference values for energy and nutrient intake [34].

### 2.3. Determination of Mineral Content

Analysis of mineral content in the samples of fillet, spine and bones of carp was performed according to the methods described by Skałecki et al. [13] in triplicate. Briefly, 8 metallic elements (K, Na, Ca, Mg, Zn, Fe, Mn and Cu) were analyzed by flame atomic absorption spectrometry (FAAS; air-acetylene flame) using a Varian AA280FS Fast Sequential Atomic Absorption Spectrometer (Varian Australia Pty Ltd., Mulgrave, Australia). The content of elements was expressed in mg kg^−1^ wet weight of samples.

### 2.4. Determination of Heavy Metals (Cd, Pb, Hg)

The concentration of Cd and Pb in the digest solution of spine, bones and fillet were determined by inductively coupled plasma mass spectrometry (Varian MS-820ICP Mass Spectrometer; Varian, Belrose, Australia). The operating parameters were as described by Kędzierska-Matysek et al. [35]. During the analysis, the following detection limits (LOD) were used: for Cd 0.004 mg/kg and for Pb 0.005 mg/kg. Research quality control was used by measuring a blank sample, a duplicate sample and Standard Reference Material 1577c Bovine Liver (NIST, Gaithersburg, MD, USA). The content of Cd and Pb was determined using the standard curve method. Ultra Scientific standards (purity of 99.999%) were used for analysis. The analytical accuracy and precision were within 5% for both metals. The results were expressed in mg kg^−1^ of fresh weight.

The Hg content was measured by means of the AMA 254 atomic absorption spectrometer according to the method presented by Kędzierska-Matysek et al. [35].

### 2.5. Assessment of Mineral Concentration in Relation to Nutrient Requirements

The content of minerals (K, Na, Ca, Mg, Fe, Cu, Zn, Mn) was used to assess the percentage coverage of the recommended dietary reference values (DRVs) for children and adults with 10 g of carp spine and bones and 100 g of fillet. The amounts of 10 g carp spine and bones were chosen, as portion included 10 g of fish powder in 100 g of food was found to be highly acceptable by consumers [36,37]. The population reference intake (PRI) and adequate intake (AI) suggested by the European Food Safety Authority [38] were adopted for calculation.

### 2.6. Statistical Analysis

The statistical calculations were made using STATISTICA 13 software (TIBCO Software Inc., Palo Alto, CA, USA)). The normality and the homogeneity of data variance were tested using the Kolmogorov–Smirnov test and Levene’s F-test, respectively. Data of proximate composition and element concentration with a normal distribution were analyzed using one-way analysis of variance (ANOVA), then to compare means between fish parts (fillet, spine and fish bones) Tukey’s (HSD) test was used. The nonparametric data (for calcium, zinc and lead) was verified by the Kruskal–Wallis test (comparison of many independent groups). Differences between means at confidence levels of 95% and 99% (*p* < 0.05 and *p* < 0.01, respectively) were considered statistically significant.

## 3. Results and Discussion

### 3.1. Proximate Composition

The chemical composition of carp parts differed significantly (*p* < 0.01) (Table 1). The highest average percentage of moisture was found in the fillet, lower in the spine and the lowest in fish bones (all means differ at *p* < 0.01). Water was the main constituent of carp fillet and spine. The moisture content in the fillet of *C. carpio* was similar to the values reported by other authors in carp [39], grass carp [40], bighead carp [41] and Prussian carp [42]. The water content observed in carp bone tissues was in accordance with the results obtained by Maktoof et al. [43] in the bones of carp (*Cyprinus carpio*) from the Euphrates River (46.40–53.6%) and by Rosmawati et al. [44] in the bones of snakehead fish (*Channa striata*) (43.19%). Li et al. [45] reported that in Asian carps (bighead carp, black carp, common carp, grass carp and silver carp) this parameter ranged from 48.76% to 66.68%. Similar water content (51–55%) was also found in bones of some marine fishes (*Pseudotolithus typus* and *Pseudotolithus elongate)* [46].

Protein content differed significantly (*p* < 0.01) among the examined carp tissues (Table 1) with the highest level in bones and lower in fillet and spine. The protein percentage found in muscle tissue in the present study was similar to those observed by Vasconi et al. [47] in the meat of freshwater fish (18–20%) and by other authors in the *Cyprinidae* family species [40,41,42]. The concentration of protein in carp spine and bones in our study was 16.5% and 21.0%, respectively. Rosmawati et al. [44] found the protein content in the bones of freshwater *C. striata* to be on the level of 15.49%. The percentage of protein in Asian carp bones reported by Li et al. [45] was from 14.57% to 18.11%, which is in accordance with our result.

The level of lipids was significantly (*p* < 0.01) higher in the bones than in the spine and fillet (Table 1). This is in accordance with Li et al. [45], who reported that in Asian carp, lipid content was from 1.27% to 13.27% in bones and from 0.12% to 8.58% in flesh. Lipid levels in fish muscle tissue are shown to range from less than 1% to more than 30% [48]. According to Toppe et al. [11], the fatty fish species store lipids in muscle tissue and bones, whereas the liver is the main organ where fat is deposited in lean fish. Taking into account the fat content in muscle tissue, analyzed individuals can be, in Polish practice, qualified as a medium fat species (2–7% of lipids). In turn, lipid content in carp skeleton was between 6.4% (spinal column) and 11.5% (fish bones). These values are slightly higher compared to the data obtained by Maktoof et al. [43] in the bones of *C. carpio* (5.9–7.00%) or by Rosmawati et al. [44] in snakehead fish bones (4.19%). Despite some possible variations in methods of sample preparation, many factors, including food composition, food and feeding habits, feeding rate, age, size, sex, habitat, genetic features and season/migration may determine fish body composition [49] and be responsible for potential differences in results obtained in different studies.

The levels of ash were significantly (*p* < 0.01) higher in inedible parts than in the fillet (Table 1), as expected. Carp spine was characterized by a high level of ash (21.8%), but the highest was observed in carp bones, where it was the main ingredient of the tissue (36.2%). Our results are in accordance with the data obtained by other authors in the bone tissues of freshwater (*Channa striata*) (32.05%) [44], as well as seawater fish (*Pseudotolithus typus* — 39.30% [46], *Sardinella gibbose* — 27.20 g/100 g, *Clupeonella engrauliformis* — 31.23 g/100 g, *Stolephorus indicus* — 23.17 g/100 g [50]). The content of ash in carp fillet observed in our study (1.2%) was typical for cyprinids [39,40,41,42].

Due to the variances in the content of major components, the energy value of carp bones was higher (*p* < 0.01) compared to the spine and the fillet (by 264 kJ and 305 kJ, accordingly) (Table 1). The energy value of 100 g fillet was in the range typical for 100 g portion of freshwater fish fillets, i.e., from 400 kJ to 800 kJ [51,52,53]. The literature on the energy value of fish bones is scarce. Only Tsibizova et al. [54] analyzed this parameter in carp bone tissue obtaining the values 107.3 kcal (448 kJ) and 101.4 kcal (424 kJ) for samples before cooking and after separation of muscle tissue, respectively. These values were lower compared to our study; however, the bone tissue analyzed in the cited study included backbone, head and fins.

The average NQI values for protein (4.7, 5.3 and 6.0 for bones, spine and fillet, respectively) did not differ significantly among analyzed carp tissues (Table 1). In turn, significant (*p* < 0.05) differences were found in NQI for lipids, with higher level in carp bones (1.8) compared to carp fillet (1.3). Hansen’s NQI, provides very useful nutrient-to-calorie comparison, as it measures the amount of nutrient in a food relative to the food’s energy content, using the recommended dietary allowance for each nutrient as the reference standard, based on the consumption of 2000 kcal (8400 kJ) energy [55]. An NQI > 1 means that consumed product provides the appropriate amount of a given nutrient (in proportion to the energy supply). Hence, all analyzed carp tissues can be treated as a good source of lipids and protein and can be used to compensate for its low content in deficit products.

### 3.2. Mineral Concentration

The highest concentrations of calcium (Ca), magnesium (Mg) and manganese (Mn) were found in the bones (*p* < 0.01), then in the spine, and the lowest in the fillet of the carp (Table 2). The levels of potassium (K) and iron (Fe) were significantly (*p* < 0.01) higher in the fillet in comparison with the spine and bones, which, in turn, contained significantly (*p* < 0.01) more sodium (Na) and zinc (Zn). The concentration of copper (Cu) was similar in all parts of carp. According to Toppe et al. [11], there is a close relationship between bone ash level and macroelement concentrations. The results obtained in the present research for macro- and microelement concentrations in muscle tissue and bones of carp are similar to data reported by other authors on flesh of cyprinids [56,57,58] and bones of *C. carpio* [59]. Lower concentrations of Na and higher K in carp fillet makes this species quite a good meal for human health, especially in the case of cardiovascular disease prevention, where the Na/K ratio in food should not be more than 1 [60,61]. Asgedom et al. [59] suggested that metals may show organ-specific accumulation, which depends to a large degree on the presence of the metal ions in the water, the physiological role of each element and the tendency of an element to bind to or replace some elements in the tissue.

### 3.3. Heavy Metals

No significant differences in lead (Pb) concentration between examined fish tissues were found (Table 3). The concentration of cadmium (Cd) in the muscle tissue was significant, (*p* < 0.01) two to three times higher than in inedible parts of carp, in which, moreover, mercury (Hg) was not detected, whereas in carp meat it was on average 0.023 mg kg^−1^.

Flesh is the most important fish organ regarding accumulation of toxic compounds because it is the most consumed one [16]. On the other hand, muscle tissue usually exhibits a low accumulation potential of metals [62]. The level of Pb in muscle tissue was lower than FAO/WHO [63] and European Commission [64] maximum guidelines for fish (0.3 mg kg^−1^). Similarly, carp meat did not exceed the European Commission maximum guidelines (0.05 mg kg^−1^) in terms of Cd and Hg (0.05 mg kg^−1^) [64]. Fish and fish products available in the Polish food market are safe for consumers [65,66]. Staszowska et al. [67] found a Pb concentration of 0.043 mg kg^−1^ in the muscle tissue of carp from Polish aquaculture. Moreover, the cited authors estimated the risk associated with lead consumption by calculating the percentage BMDL (benchmark dose lower confidence limit) in a meat portion of 100 g and revealed that the safe amount of carp for an adult should not exceed 1015 g (nephrotoxic effects BMDL_10_) and 2400 g (cardiovascular disorders BMDL_01_), respectively.

The differences in heavy metal concentrations observed between the spine, bones and fillet (Table 3) might be due to the different affinity of various metals to fish tissues [68]. Cadmium is usually accumulated in the kidney, liver, gills, digestive tract and spleen, which may explain its low level in carp tissues in our study. Lead deposits in the liver, kidneys and spleen; digestive tract; and gills, but high levels of this metal are also sometimes found in bones [68]. Concentrations of Hg in flesh very often exceeds level of mercury in other organs [16], which may be the explanation why this metal in our study was found only in the carp fillet, but was not detected in the bones or the spine.

### 3.4. Mineral Concentration in Relation to Nutrient Requirements

Percentile contribution of spine, bones and fillet of *C. carpio* to PRI (population reference intake) or AI (adequate intake) for different consumer groups is presented in Table 4. As expected, carp spine and bones were characterized by high contributions to dietary reference intake of calcium. The calculation results indicate that 10 g of *C. carpio* spine met 70% of the AI for adults and 86% for children and in the case of bones, corresponding levels were 119% and 146% of the AI, respectively.

Carp fillet showed higher, compared to the spine and the bones, percentile coverage of the nutrient reference value for K, Mg, Fe and Cu. However, taking into account the European Union legislation [34], which defined ‘a significant amount minerals’ ensured by a product as at least 15% of the DRV, carp fillet covered remarkably the dietary reference value only in case of potassium for children.

Carp spine in bone provided the recommended nutrition standards for zinc to a higher extend compared to the fillet. In the case of bones, it was even two times as much as in fillet. Nevertheless, analyzing recommendation of EFSA [38] carp bones and spine could remarkably (≥15%) contribute only to the recommended intake of Zn for toddlers during the second half of the first year of life (2.9 mg/day).

Both prepared portions of fillet, as well as spine and bones, assured only 2–3% of the daily requirements for sodium. This is very important, as the global average sodium intake far exceeds the physiological requirement [69].

## 4. Conclusions

This study shows that *C. carpio* is good source of many major nutrients and essential elements. All carp tissues contain high levels of proteins and could be used as an animal protein source. Carp bone tissue, especially bones (ribs), contain high levels of calcium and can be considered as the calcium fortifier for food products in human diets. Percentile coverage of nutrient reference value for Ca by carp bone tissues may exceed 100%. The content of heavy metals did not exceed limits given by the FAO and the European Commission. Then, we can conclude that all the analyzed parts of *C. carpio* species can be recommended for both animal and human consumption and health. However, due to the limited number of samples and their character (market samples), this study may not provide fully representative characteristics of *C. carpio* tissues; therefore further research in this area is needed to verify our findings.

## Figures and Tables

**Table 1:** Proximate composition (%), energy (kJ/100 g) and NQI values of carp (*C. carpio*) spine, bones and fillet (mean ± standard deviation).

Specification	Spine (*n* = 15)	Bones (*n* = 15)	Fillet (*n* = 15)
Moisture	56.0 ^B^ ± 0.8	31.8 ^A^ ± 0.2	77.8 ^C^ ± 0.5
Protein	16.5 ^A^ ± 0.9	21.0 ^B^ ± 1.9	17.0 ^A^ ± 0.3
Lipids	6.4 ^A^ ± 1.6	11.5 ^B^ ± 2.2	5.1 ^A^ ± 0.6
Ash	21.8 ^B^ ± 0.8	36.2 ^C^ ± 0.6	1.2 ^A^ ± 0.1
Energy	519 ^A^ ± 46	782 ^B^ ± 99	478 ^A^ ± 18
NQI			
Protein	5.3 ± 0.8	4.7 ± 0.3	6.0 ± 0.6
Lipids	1.5 ^ab^ ± 0.3	1.8 ^b^ ± 0.3	1.3 ^a^ ± 0.0

^a, b^ Means with different letters in rows differ statistically significantly at *p* < 0.05, ^A, B, C^ Means with different letters in rows differ statistically significantly at *p* < 0.01. NQI, Nutritional Quality Index.

**Table 2:** Concentration of macro- and microelements (mg kg^−1^) in carp (*C. carpio*) spine, bones and fillet (mean ± standard deviation).

Element	Spine (*n* = 15)	Bones (*n* = 15)	Fillet (*n* = 15)
Macroelements			
K	1267 ^A^ ± 90	1320 ^A^ ± 250	4005 ^B^ ± 351
Na	2947 ^B^ ± 184	3047 ^B^ ± 451	401 ^A^ ± 20
Ca	68,433 ^B^ ± 14,090	116,463 ^C^ ± 15,190	334 ^A^ ± 88
Mg	836 ^B^ ± 202	1427 ^C^ ± 295	301 ^A^ ± 26
Microelements			
Fe	1.6 ^A^ ± 0.9	2.7 ^A^ ± 0.1	6.9 ^B^ ± 2.3
Cu	0.9 ± 0.1	1.4 ± 0.2	1.1 ± 0.3
Zn	43.6 ^B^ ± 21.2	54.3 ^B^ ± 11.2	2.8 ^A^ ± 0.9
Mn	5.2 ^B^ ± 1.3	8.1 ^C^ ± 1.5	0.9 ^A^ ± 0.2

^A, B, C^ Means with different letters in rows differ statistically significantly at *p* < 0.01. K, Potassium; Na, Sodium; Ca, Calcium; Mg, Magnesium; Fe, Iron; Cu, Copper; Zn, Zinc; Mn, Manganese.

**Table 3:** Concentration of heavy metals (mg kg^−1^) in carp (*C. carpio*) spine, bones and fillet (mean ± standard deviation).

Element	Spine (*n* = 15)	Bones (*n* = 15)	Fillet (*n* = 15)
Hg	–	–	0.023 ± 0.010
Pb	0.107 ± 0.064	0.109 ± 0.021	0.035 ± 0.017
Cd	0.002 ^A^ ± 0.000	0.003 ^A^ ± 0.001	0.006 ^B^ ± 0.001

^A, B^ Means with different letters in rows differ statistically significantly at *p* < 0.01. Hg, Mercury; Pb, Lead; Cd, Cadmium.

**Table 4:** Estimated elemental contribution (%) of carp (*C. carpio*) spine (10 g), bones (10 g) and fillet (100 g) to dietary reference value for various population groups.

Element	Population Group	mg per Day *	Dietary Reference Value (DRV)	Spine	Bones	Fillet
K	women	3500 ^a^	%AI	0	0	11
	men	3500 ^a^		0	0	11
	children	1450 ^b^		1	1	28
Na	women	2000 ^a^	%AI	2	2	3
	men	2000 ^a^		2	2	3
	children	1500 ^b^		2	3	3
Ca	women	975 ^c^	%PRI	70	119	3
	men	975 ^c^		70	119	3
	children	800 ^d^		86	146	4
Mg	women	300 ^a^	%AI	3	5	10
	men	350 ^a^		2	4	9
	children	230 ^e^		4	6	13
Fe	women	16 ^a^	%PRI	0	0	4
	men	11 ^a^		0	0	6
	children	9 ^f^		0	0	8
Cu	women	1.3 ^a^	%AI	1	1	9
	men	1.6 ^a^		1	1	7
	children	1 ^e^		1	1	11
Zn	women	10.125 ^g^	%PRI	4	5	3
	men	12.85 ^g^		3	4	2
	children	6.45 ^b^		7	8	4
Mn	women	3 ^a^	%AI	2	3	3
	men	3 ^a^		2	3	3
	children	1.25 ^b^		4	6	7

K, Potassium; Na, Sodium; Ca, Calcium; Mg, Magnesium; Fe, Iron; Cu, Copper; Zn, Zinc; Mn, Manganese. * DRV—Dietary Reference Value (mg per day); PRI—population reference intake; AI—an adequate intake; source for nutrients requirements EFSA [38]. ^a^ DRV for adults, i.e., non-lactating, non-pregnant, premenopausal women and men aged 18 and older (≥18). ^b^ DRV for children (male and female) aged 4–10 years—average calculated from the standards for the age group 4–6 years and 7–10 years. ^c^ DRV for adults (male and female) aged 18 and older (≥18)—average calculated from the standards for the age group 18–24 years and ≥25 years. ^d^ DRV for children (male and female) aged 4–10 years. ^e^ DRV for children (male and female) aged 3–9 years. ^f^ DRV for children (male and female) aged 4–10 years—average calculated from the standards for the age group 4–6 years and 7–10 years for boys and group 4–6 years and 7–11 years for girls. ^g^ DRV is given as the average calculated from the standards for women (7.5–12.7 mg/day) and men (9.4–16.3 mg/day).

## Data Availability

The data are available from the authors upon reader’s request.
